# Chalcone-9: a novel inhibitor of the JAK-STAT pathway with potent anti-cancer effects in triple-negative breast cancer cells

**DOI:** 10.1007/s43440-025-00721-w

**Published:** 2025-04-09

**Authors:** Song-Hee Lee, Haeri Lee, Yong-Jin Kwon, Seul-Ki Kim, Eun-Bi Seo, Jie Ohn Sohn, Byung-Hak Kim, Jung-Youl Park, Sang-Kyu Ye

**Affiliations:** 1 Department of Biomedical Sciences and Pharmacology, Seoul National University College of Medicine, 103 Daehak-Ro, Jongno-Gu, Seoul, 03080 Republic of Korea; 2 Ischemic/Hypoxic Disease Institute, Seoul National University College of Medicine, Seoul, 03080 Republic of Korea; 3 Department of Cosmetic Science, Kyungsung University, Busan, 48434 Republic of Korea; 4 Wide River Institute of Immunology, Seoul National University, Hongcheon, Gangwon-Do 25159 Republic of Korea; 5 Medience Co. Ltd., Chuncheon, Gangwon-Do 24232 Republic of Korea; 6 Glocal University Project Group, Andong National University, Andong, Gyeongsangbuk-Do 36729 Republic of Korea; 7 Biomedical Science Project (BK21PLUS), Seoul National University College of Medicine, Seoul, 03080 Republic of Korea; 8 Neuro-Immune Information Storage Network Research Center, Seoul National University College of Medicine, Seoul, 03080 Republic of Korea

**Keywords:** Triple-negative breast cancer (TNBC), Anti-cancer activity, (E)-4-(3-(2-(benzyloxy)-6-hydroxyphenyl)-3-oxoprop-1-en-1-yl)benzoic acid (chalcone-9), JAK-STAT pathway, Apoptosis

## Abstract

**Background:**

Breast cancer remains the leading cause of cancer incidence and mortality among women worldwide, with triple-negative breast cancer (TNBC) posing significant treatment challenges. The dysregulation of the Janus kinase/signal transducer and activator of transcription (JAK/STAT) pathway contributes to tumor progression, making it a potential therapeutic target. Chalcones, known for their diverse biological activities, including anti-cancer effects, hold promise for drug development. This study explores the anti-cancer activity of (E)-4-(3-(2-(benzyloxy)-6-hydroxyphenyl)-3-oxoprop-1-en-1-yl)benzoic acid (chalcone-9), a novel chalcone derivative.

**Methods:**

The cytotoxic effects of chalcone-9 were evaluated in breast cancer cell lines, including TNBC lines MDA-MB-231 and MDA-MB-468. Western blotting and qRT-PCR were used to analyze the impact on JAK1, JAK2, STAT1, and STAT3 activation and their downstream gene expression. In silico molecular docking analysis was conducted to determine whether chalcone-9 can interact with JAK1 and JAK2. A wound healing assay was used to observe the effect of chalcone-9 on tumor cell migration, and flow cytometry was employed to analyze whether chalcone-9 inhibits tumor cell cycle progression and induces apoptosis. The expression of apoptosis markers was also assessed.

**Results:**

Chalcone-9 exhibited dose-dependent cytotoxicity in breast cancer cell lines, with TNBC cells showing higher sensitivity. Chalcone-9 effectively inhibited the activation of JAK1, JAK2, STAT1, and STAT3, outperforming conventional JAK/STAT inhibitors. The structure of chalcone-9 was confirmed to stably interact with JAK1 and JAK2 proteins. It also suppressed STAT1 and STAT3 target gene expression, reduced tumor cell migration, and induced apoptosis, as evidenced by PARP and caspase cleavage and decreased survivin levels.

**Conclusions:**

Chalcone-9 demonstrates significant anti-cancer activity, particularly against TNBC. By targeting the JAK/STAT pathway and promoting apoptosis, chalcone-9 emerges as a promising therapeutic candidate for aggressive breast cancers.

**Graphical abstract:**

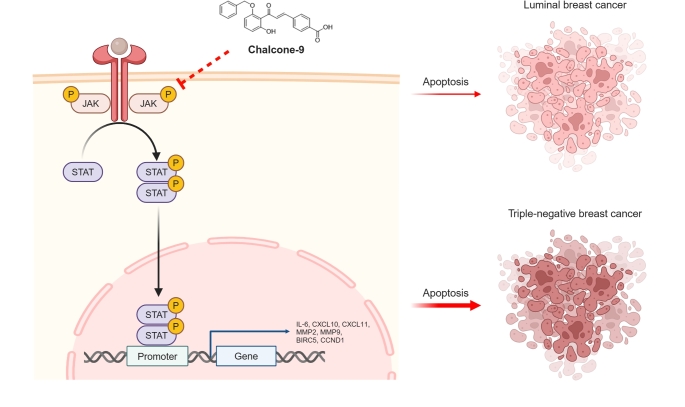

**Supplementary Information:**

The online version contains supplementary material available at 10.1007/s43440-025-00721-w.

## Introduction

Breast cancer is the type of cancer that has the highest incidence and mortality rates among women worldwide. Due to its aggressive and metastatic nature, achieving a full recovery of patients remains significantly challenging [1, 2]. Despite advances in breast cancer research, the heterogeneity of breast cancer makes it difficult to administer appropriate treatments. Therefore, it is crucial to classify breast tumors into distinct subtypes based on their gene expression profiles. Conventionally, based on the expression features of three representative receptors: estrogen receptor (ER), progesterone receptor (PR), and human epidermal receptor 2 (HER2), breast cancers have been classified into luminal A, luminal B, HER2-positive, and triple-negative breast cancer (TNBC) subtypes. Among these, the TNBC subtypes, which lack ER, PR, and HER2 expression, are the most aggressive type with a poor prognosis [3, 4]. Because the TNBC subtypes do not express the three receptors, there are limitations to therapies such as trastuzumab and pertuzumab (; targeting HER2) or tamoxifen (; targeting ER), which are commonly used in breast cancer [5, 6]. Therefore, new therapeutic strategies are needed to overcome these limitations.

The Janus kinase/signal transduction and transcription activation (JAK/STAT) pathway is crucial in regulating diverse cellular functions, such as proliferation, survival, migration, and immune responses, by controlling target gene transcription [1, 2]. When cytokines or growth factors bind to their receptors, JAK kinases are activated and then phosphorylate the cognate receptor and STATs. Subsequently, phosphorylated STATs form homodimers or heterodimers, translocate into the nucleus, and modulate gene transcription by binding to DNA. Despite its essential functions, the constitutive activation of the JAK-STAT signaling pathway is implicated in various diseases, including autoimmune disorders, inflammation, and cancers. Specifically, in cancers, excessively activated JAK-STAT signaling promotes tumor growth, metastasis, immune evasion, drug resistance, etc [7–10]. For these reasons, it is important to develop compounds that effectively inhibit the JAK-STAT signaling pathway. To achieve this goal, we have researched the inhibition of the JAK-STAT signaling in cancer and other diseases [11–17]. Our previous studies reported that treatments with natural plant extracts and novel chemicals display anti-inflammatory or anti-cancer effects by inhibiting the JAK-STAT signaling.

Chalcones consist of two aromatic rings linked by a 3-carbon unsaturated α, β-carbonyl system and are common in many natural products. Chalcone compounds, with a versatile scaffold that can be easily modified, can bind to different molecular targets by adding various functional groups (aryls, carboxyls, hydroxyls, phenyl, etc.). This ability allows them to exhibit a broad spectrum of biological activities, making chalcone compounds valuable templates for developing novel anti-cancer agents [18–20].

We previously reported a variety of chemically synthesized chalcone derivatives [21, 22]. In previous studies, we evaluated and optimized the anti-melanogenic activity of these derivatives. On the other hand, this study aimed to identify the chalcone derivative that functions as a JAK-STAT pathway inhibitor and demonstrate its anti-cancer activity. We observed significant anti-cancer effects of (E)-4-(3-(2-(benzyloxy)-6-hydroxyphenyl)-3-oxoprop-1-en-1-yl)benzoic acid (chalcone-9) among other chalcone derivatives in breast cancer cells. Chalcone-9 is one of the chalcone derivatives reported earlier [22]. We discovered that chalcone-9 inhibited the activation of the JAK-STAT signaling pathway, decreased the mRNA expression of STAT1 and STAT3 target genes, and impaired cancer cell functions in breast cancer cells. Consequently, chalcone-9 induced cancer cell apoptosis. These results suggest that chalcone-9 is a potent anti-cancer agent in breast cancer.

## Materials and methods

### Compounds and reagents

All treated chalcone derivatives, including chalcone-9, were chemically synthesized by aldol condensation as previously described [22]. The chalcone derivatives used in this study are as follows; (E)-1-(2-(benzyloxy)-6-hydroxyphenyl)-3-(4-ethylphenyl)prop-2-en-1-one (Chalcone-6), (E)-1-(2-(benzyloxy)-6-hydroxyphenyl)-3-(4-chlorophenyl)prop-2-en-1-one (Chalcone-7), (E)-1-(2-(benzyloxy)-6-hydroxyphenyl)-3-(4-methoxyphenyl)prop-2-en-1-one (Chalcone-8), (E)-4-(3-(2-(Benzyloxy)-6-hydroxyphenyl)-3-oxoprop-1-en-1-yl)benzoic acid (Chalcone-9). Cells were treated with 0.1% dimethyl sulfoxide (DMSO) as a vehicle or chalcone derivatives. Tamoxifen (cat. T5648, Sigma-Aldrich, St. Louis, MO, USA) and AG490 (cat. Sc-202046A, Santa Cruz Biotechnology, Dallas, TX, USA) were used in this study. Primary antibodies specific for pY-JAK1 (Y1034/1035) (cat. #3331), JAK1 (cat. #3332), pY-JAK2 (Y1007/1008) (cat. #3776), JAK2 (cat. #3230), pY-JAK3 (Y980/981) (cat. #5031), JAK3 (cat. #8863), pY-TYK2 (Y1054/1055) (cat. #68790), TYK2 (cat. #14193), pY-STAT1 (Y701) (cat. #9167), pS-STAT1 (S727) (cat. #8826), STAT1 (cat. #9172), pY-STAT3 (Y705) (cat. #9145), pS-STAT3 (S727) (cat. #9134), STAT3 (cat. #30835), pY-STAT5 (Y694) (cat. #9359), STAT5 (cat. #94205), survivin (cat. #2808), caspase-3 (cat. #9662), caspase-9 (cat. #9502) and PARP (cat. #9542) were purchased from Cell Signaling Technology (Danvers, MA, USA). Anti-α-tubulin antibody (cat. #A01080, Abbkine, Wuhan, China) and anti-GAPDH antibody (cat. ABc-2003, AbClon Inc., Seoul, Republic of Korea) were used. Anti-rabbit IgG (cat. ADI-SAB-300-J, Enzo Life Sciences, Farmingdale, NY, USA) and anti-mouse IgG (cat. ADI-SAB-100-J, Enzo Life Sciences) were used for the secondary antibodies.

### Cell culture

The human breast cancer cell lines MCF-7, T47D, MDA-MB-231, and MDA-MB-468 were obtained from the American Type Culture Collection (ATCC, Manassas, VA, USA). Cells were cultured in Dulbecco’s modified essential medium (DMEM; Hyclone, Logan, UT, USA) supplemented with 10% fetal bovine serum (FBS; Hyclone) and 1% penicillin/streptomycin (Capricorn Scientific, Ebsdorfergrund, Germany), and maintained in a humidified incubator with 5% CO_2_ at 37 °C.

### Cell viability assay

Cell viability was estimated by CCK-8 (Cell Counting Kit-8; Dongin-LS, Seoul, Republic of Korea) assay. For CCK-8 assay, 8 × 10^3^ −1 × 10^4^ cells were seeded into 96-well plates and given 24 h to attach. Cells were treated with 0.1% DMSO as a vehicle or various concentrations of chalcone-9 for 24 h. After treatment, the conditioned medium was discarded gently, and cells were incubated with 100 µl/well of the CCK-8-contained medium (1:100) for 2–4 h at 37 °C. The absorbance was measured at the wavelength of 450 nm using a microplate reader (Tecan, Männedorf, Switzerland).

### Western blotting

Cell lysates were prepared using Triton X-100 lysis buffer (150 nM NaCl, 20 mM Tris, 1% Triton X-100, 1% sodium deoxycholate, 0.1% sodium dodecyl sulfate (SDS), 10 mM ethylenediaminetetraacetic acid (EDTA)) with protease/phosphatase inhibitors (NaF, Na_3_VO_4_, Leupeptin and phenylmethylsulfonyl fluoride (PMSF)). Bradford assay was used to measure the unknown protein concentrations. The lysate of proteins was separated via SDS-polyacrylamide gel electrophoresis and then transferred to nitrocellulose membranes (GE Healthcare, Chicago, IL, USA). Membranes were incubated with blocking buffer (Tris-buffered saline (TBS) with 5% skimmed milk (LPS solution, Daejeon, Republic of Korea) and 0.1% tween 20 (LPS solution)) for 1 h at room temperature followed by incubating at 4 °C overnight with primary antibodies (1:1000). After washing, the membranes were treated with horseradish peroxidase-conjugated secondary antibodies (1:10000) (Enzo Life Science, Farmingdale, NY, USA) for 1 h, rewashed, and then the signal was visualized using an ECL detection kit (BioMax, Seoul, Republic of Korea). Densitometric quantification of the band intensity was performed by ImageJ software (NIH, Bethesda, MD, USA) and normalized to the loading control, which is α-tubulin or GAPDH.

### In silico molecular docking

The interaction between the compound and target proteins was analyzed using the Autodock-VINA program version 1.2.3. The structures of the target proteins used in the analysis were obtained from the PDB database and are as follows: JAK1 (PDB: 5KHW), and JAK2 (PDB: 2B7A). The compound (; chalcone-9) was optimized using ChemBio3D. Following this, it was converted into PDBQT format using Autodock tools.

### Quantitative real-time PCR (qRT-PCR)

Total RNA was isolated using the RNAiso Plus reagent (Takara, Shiga, Japan), and cDNA was synthesized using the ReverTra Ace qPCR RT Master Mix (Toyobo, Osaka, Japan). The mRNA expression level of individual target genes was detected using the BlasTaq 2X qRT-PCR MasterMix (Applied Biological Materials, Richmond, Canada). Quantitative real-time PCR (qRT-PCR) was performed on the CFX Connect Real-Time PCR Detection System (Bio-Rad Laboratories, Hercules, CA, USA) with the following PCR conditions: 95 °C for 10 min, followed by 40 cycles of 95 °C for 10 s, 60 °C for 10 s, and 72 °C for 20 s. The raw data were analyzed by comparative Ct quantification and normalized to internal control GAPDH. Primers for IL-6 (QT00083720), CXCL10 (QT01003065), CXCL11 (QT00233387), MMP2 (QT00088396), MMP9 (QT00040040), BIRC5 (QT01679664), CCND1 (QT00495285) and GAPDH (QT01192646) were purchased from Qiagen (Hilden, Germany).

### Wound healing assay

Cell migration was assessed by a wound healing assay. 4 × 10^5^ cells were seeded into 12-well plates, and scratch wounds were made using sterile 200 μl pipette tips when cells reached 80 – 90% of confluence. The detached cells were washed out with phosphate-buffered saline (PBS), and 1 ml of chalcone-9-contained medium with 2% FBS was treated for 24 h. The wound closure was monitored every 6 h using an Incucyte Live-Cell Analysis System (Sartorius, Göttingen, Germany), and the wound area was analyzed using ImageJ software. The migration density (%) represents the area covered by migrating cells over time within the initial scratch wound area at 0 h. The quantification method is as follows.

$$\begin{aligned}&{\rm{Migration}}{\mkern 1mu}\:{\rm{density}}{\mkern 1mu}\:({\rm{\% }}) \cr&\quad= {\left\{ {\begin{aligned}&\{ {\rm{Initial}}{\mkern 1mu}\:{\rm{scratch}}{\mkern 1mu}\:{\rm{wound}}\:({\rm{t}}_{0}) \cr&\quad- {\rm{Scratch}}{\mkern 1mu}\:{\rm{wound}}{\mkern 1mu}\:{\rm{at}}{\mkern 1mu}\:{\rm{time}}{\mkern 1mu}\:{\rm{X}}{\mkern 1mu}\:{\rm{h}}\:({\rm{t_{X}}})\} \end{aligned}} \right\} \over {{\rm{Initial}}{\mkern 1mu}\:{\rm{scratch}}{\mkern 1mu}\:{\rm{wound}}\:({\rm{t}}_{0})}} \times 100\end{aligned}$$

### Cell cycle and apoptosis assessment by flow cytometry

For cell cycle assay, cells were fixed in cold 70% ethanol and washed with PBS and 1× binding buffer (BD Bioscience, Franklin Lakes, NJ, USA). Then, cells were treated with 100 μg/ml RNase A (Real Biotech Corporation, Taipei, Taiwan) for 30 min at 37 °C and stained with 2.5 μg/ml propidium iodide (PI) (BD Bioscience) for 30 min at room temperature. For cell apoptosis assay, cells were stained with FITC Annexin-V and PI kit (BD Bioscience) according to the manufacturer’s protocol. Stained cells were analyzed using BD LSRFortessa (BD Bioscience) and FlowJo 10.7 software (BD Bioscience).

### Statistical analysis

All results were analyzed using Microsoft Excel 2019 or GraphPad Prism 8 (GraphPad Software, Inc., San Diego, CA, USA) and were presented as the means ± standard deviation (SD) from three independent samples. One-way analysis of variance (ANOVA) was conducted to compare multiple groups, followed by Tukey’s post hoc test for pairwise comparisons and Dunnett’s post hoc test for comparison between the experimental and control groups. For comparisons between two groups, an unpaired Student’s *t*-test was used. A *p*-value less than 0.05 was considered statistically significant. *p < 0.05, **p < 0.01, ***p < 0.001.

## Results

### Chalcone-9 exhibits the most significant inhibition of JAK-STAT activation in breast cancer cells

**Fig. 1:**
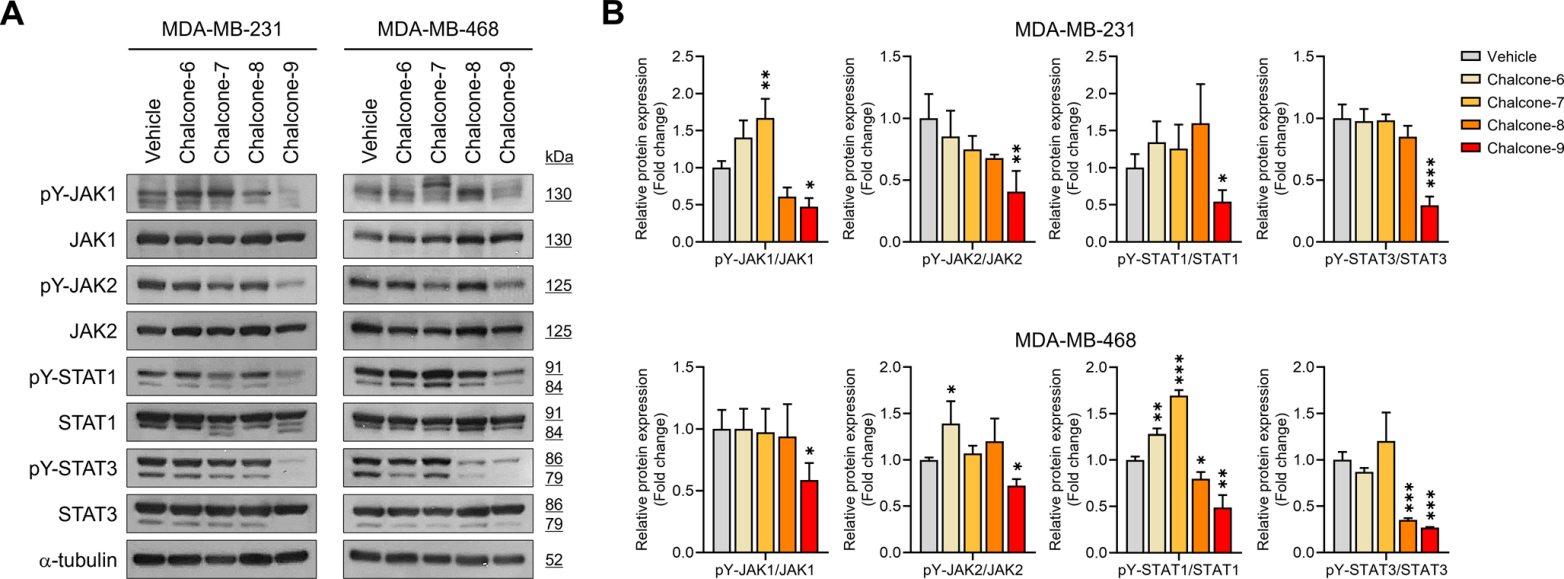
Comparison of the inhibitory effect of the chalcone derivatives on the JAK-STAT pathway. **A** Western blot analysis for JAK1, JAK2, STAT1, STAT3 phosphorylation, and α-tubulin following treatment with 50 μM of chalcone derivatives for 6 h in MDA-MB-231 and MDA-MB-468 cells. **B** quantification of the relative pY-JAK1/JAK1, pY-JAK2/JAK2, pY-STAT1/STAT1, and pY-STAT3/STAT3 protein expression levels in MDA-MB-231 and MDA-MB-468 cells. Data were shown as mean ± SD (*n* = 3). Statistical significance is indicated as *p < 0.05, **p < 0.01, ***p < 0.001; compared with the vehicle-treated group. All statistical analyses were performed using one-way ANOVA followed by Dunnett’s post hoc test. JAK; janus kinase, pY; phosphotyrosine, SD; standard deviation, STAT; signal transducer and activator of transcription

To identify a chalcone derivative that most effectively inhibits the JAK-STAT pathway, we compared four chalcone derivatives, each containing the common 2-(benzyloxy)-6-hydroxyphenyl group within the chalcone derivatives we reported (Fig. S1). Given that the hydroxyl functional group on the chalcone structure is associated with enhanced anti-cancer activity [23], we selected these four derivatives for our study. Chalcone-9 most significantly inhibited the phosphorylation of JAK1, JAK2, STAT1, and STAT3 in MDA-MB-231 and MDA-MB-468 cells (Fig. 1A and B). Chalcone-8 appeared to have an inhibitory effect on the JAK-STAT pathway compared to the other two derivatives, chalcone-6 and chalcone-7. However, its impact was less potent than that of chalcone-9 and showed no significant cytotoxic effect on breast cancer cells (Fig. S2). Since our objective was to select a compound exhibiting cytotoxic effects against breast cancer cells, as well as to identify a novel inhibitor of the JAK-STAT pathway, these results indicate that chalcone-9 is the most suitable candidate for further research as an anti-cancer agent.

### Chalcone-9 has the potential to be an anti-cancer drug against aggressive breast cancer cells

**Fig. 2:**
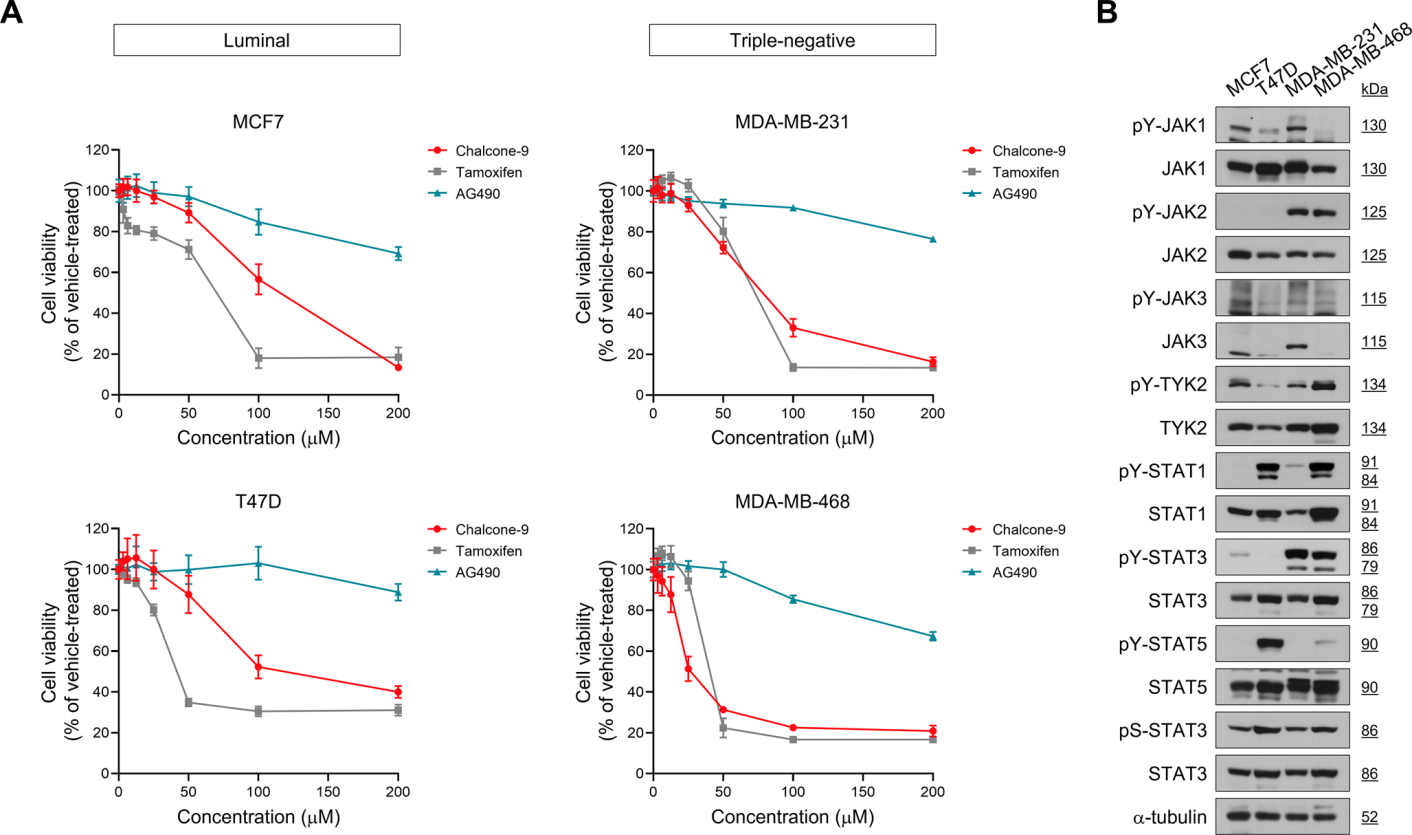
Effect of chalcone-9 on cell viability in various breast cancer cell lines. **A** cell viability and IC_50_ were measured by CCK-8 assay in breast cancer cell lines; MCF7, T47D, MDA-MB-231, and MDA-MB-468 cells. Cell viability is represented as % control compared with the vehicle-treated group. Data were shown as mean ± SD (*n* = 8). **B** Western blot analysis for JAK-STAT signaling and α-tubulin in lysates from MCF7, T47D, MDA-MB-231, and MDA-MB-468 cells. JAK; janus kinase, pY; phosphotyrosine, pS; phosphoserine, SD; standard deviation, STAT; signal transducer and activator of transcription, TYK; tyrosine kinase

To evaluate the anti-cancer properties of chalcone-9 against breast cancer cells, we examined the cell viability of various human breast cancer cell lines under a gradient of chalcone-9 concentrations. The cytotoxic effect of chalcone-9 was dose-dependent in all breast cancer cell lines we tested. In MDA-MB-231 cells, chalcone-9 treatment reduced cell viability to 72.2% ± 2.9% at 50 μM and 33.0% ± 4.4% at 100 μM, with the IC_50_ value of 78.3 μM. In MDA-MB-468 cells, chalcone-9 treatment decreased the cell viability to 31.2% ± 1.3% at 50 μM and 22.6% ± 1.3% at 100 μM, with the IC_50_ value of 26.7 μM. However, in MCF7 cells and T47D cells, even with 100 μM chalcone-9 treatment, cell viability was suppressed to 56.6% ± 7.4% and 52.2% ± 5.7%, respectively, and IC_50_ values were 115.2 μM and 118.1 μM. (Fig. 2A). These data indicate that MDA-MB-231 and MDA-MB-468 cells, representing TNBC subtype with a poorer prognosis, are more sensitive to chalcone-9 compared to luminal breast cancer subtype, such as MCF7 and T47D.

To compare the anti-cancer effects of chalcone-9 with tamoxifen, an FDA-approved drug used for breast cancer treatment, and AG490, a selective JAK2 inhibitor frequently utilized in research, we evaluated their cytotoxic effects on breast cancer cells. The anti-cancer activity of tamoxifen, which inhibits the cell viability of breast cancer cell lines, was found to be stronger than that of chalcone-9. The IC_50_ values of tamoxifen were 69.9 μM in MCF7 cells, 41.6 μM in T47D cells, 72.7 μM in MDA-MB-231 cells, and 40.4 μM in MDA-MB-468 cells. Although tamoxifen exhibited greater cytotoxic activity against breast cancer cells compared to chalcone-9, its non-selective high cytotoxicity across all cell types could lead to severe side effects in breast cancer patients by affecting normal cells during treatment. In contrast, the selective inhibition of cell viability by chalcone-9, particularly in aggressive TNBC cells, represents a significant advantage. Furthermore, AG490 showed significantly lower cytotoxic effects overall compared to both chalcone-9 and tamoxifen.

We investigated the expression pattern of the JAK-STAT signaling pathway in these four breast cancer cell lines and observed high activation of JAK2 and STAT3 in TNBC cell lines (Fig. 2B).

Based on the cytotoxic effects of chalcone-9 and the expression patterns of the JAK-STAT signaling pathway in each breast cancer cell line, our next objective was to explore more precisely the inhibitory effect of chalcone-9 on the JAK-STAT signaling pathway.

### Chalcone-9 inhibits JAK-STAT activation in breast cancer cells

**Fig. 3:**
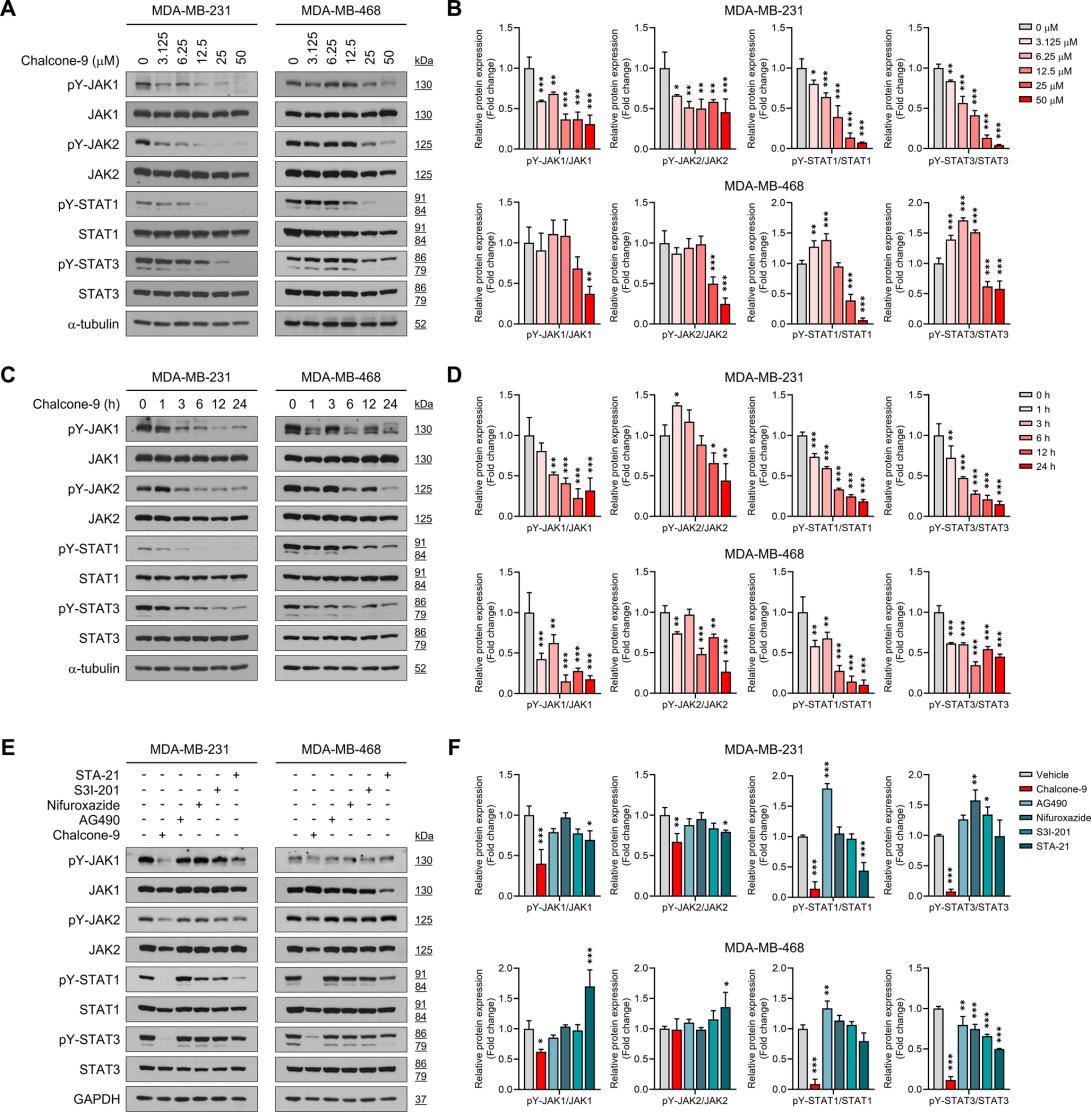
The inhibitory effect of chalcone-9 on JAK1, JAK2, STAT1, and STAT3 phosphorylation. **A**, **C** Western blot analysis for JAK1, JAK2, STAT1, STAT3 phosphorylation, and α-tubulin by a dose-dependent manner (**A**) and time-dependent manner (**C**) in MDA-MB-231 and MDA-MB-468 cells. **B**, **D** quantification of the relative pY-JAK1/JAK1, pY-JAK2/JAK2, pY-STAT1/STAT1, and pY-STAT3/STAT3 protein expression levels by a dose-dependent manner (B) and time-dependent manner (**D**) in MDA-MB-231 and MDA-MB-468 cells. Data were shown as mean ± SD (*n* = 3). Statistical significance is indicated as *p < 0.05, **p < 0.01, ***p < 0.001; compared with the vehicle-treated. All statistical analyses were performed using one-way ANOVA followed by Dunnett’s post hoc test. **E** Western blot analysis of JAK1, JAK2, STAT1, STAT3 phosphorylation, and GAPDH at treatment with 50 μM of chalcone-9, AG490, nifuroxazide, S3I-201, and STA-21 for 12 h in MDA-MB-231 and MDA-MB-468 cells. **F** quantification of the relative pY-JAK1/JAK1, pY-JAK2/JAK2, pY-STAT1/STAT1, and pY-STAT3/STAT3 protein expression levels, as shown in E. Data were shown as mean ± SD (*n* = 3). Statistical significance is indicated as *p < 0.05, **p < 0.01, ***p < 0.001; compared with the vehicle-treated group. All statistical analyses were performed using one-way ANOVA followed by Dunnett’s post hoc test. JAK; janus kinase, pY; phosphotyrosine, SD; standard deviation, STAT; signal transducer and activator of transcription

To comprehensively understand the impact of chalcone-9 on the JAK-STAT signaling pathway, we treated MDA-MB-231 and MDA-MB-468 cells with chalcone-9 at various concentrations and times. The phosphorylation of JAK1, JAK2, STAT1, and STAT3 in MDA-MB-231 cells significantly decreased upon treatment with chalcone-9 at a concentration of 6.25 μM, and the reduction continued in a dose-dependent manner at 12.5, 25, and 50 μM for 24 h (Fig. 3A and B). In MDA-MB-231 cells, treatment with 50 μM of chalcone-9 for 24 h led to a reduction in relative protein expression compared to the vehicle control: pY-JAK1/JAK1 by 0.69-fold (F_5,12_ = 28.17, *p* < 0.0001), pY-JAK2/JAK2 by 0.54-fold (F_5,12_ = 8.318, *p* = 0.0005), pY-STAT1/STAT1 by 0.93-fold (F_5,12_ = 60.64, *p* < 0.0001), and pY-STAT3/STAT3 by 0.96-fold (F_5,12_ = 188.5, *p* < 0.0001). While the inhibitory effect was most significant at 50 μM of chalcone-9, this concentration was considered too high, impairing cell viability for protein-level analysis. Hence, chalcone-9 was administered at a concentration of 25 μM for the time-course treatment experiments spanning from 1 to 24 h.

In MDA-MB-231 cells, the tyrosine phosphorylation of JAK1 was sufficiently reduced after 6 h of chalcone-9 treatment, while the tyrosine phosphorylation of JAK2 decreased after 12 h of chalcone-9 treatment. Moreover, STAT1 and STAT3 tyrosine phosphorylation were substantially suppressed after 1 and 3 h of chalcone-9 treatment, respectively (Fig. 3C and D). In MDA-MB-231 cells treated for 24 h, chalcone-9 at 25 μM reduced the relative protein expression compared to the vehicle control as follows: pY-JAK1/JAK1 by 0.68-fold (F_5,12_ = 16.01, *p* = 0.0002), pY-JAK2/JAK2 by 0.56-fold (F_5,12_ = 18.66, *p* = 0.0013), pY-STAT1/STAT1 by 0.82-fold (F_5,12_ = 362.4, *p* < 0.0001), and pY-STAT3/STAT3 by 0.85-fold (F_5,12_ = 42.22, *p* < 0.0001). Also, the inhibitory effect of chalcone-9 on the tyrosine phosphorylation of JAK1, JAK2, STAT1, and STAT3 at various concentrations and time points was validated in MDA-MB-468 cells. Overall, we propose that chalcone-9 acts primarily as an inhibitor for JAK1 and JAK2, the upstream proteins of STAT3 in the JAK-STAT signaling axis. We investigated the impact of chalcone-9 on the serine phosphorylation of STAT1 and STAT3. However, there were no significant differences in their serine phosphorylation levels by chalcone-9 (Fig. S3).

Lastly, for a comparative assessment of the inhibitory efficacy of chalcone-9 on the JAK-STAT signaling pathway with other common JAK and STAT inhibitors in breast cancer cells, the representative JAK inhibitors (AG490 and nifuroxazide) and STAT3 inhibitors (S3I-201 and STA-21) were administered alongside chalcone-9 at identical concentrations and durations. In MDA-MB-231 cells, chalcone-9 was the most effective in inhibiting the activation of JAK1 (by 0.60-fold, F_5,12_ = 13.08, *p* < 0.0001), JAK2 (by 0.33-fold, F_5,12_ = 6.781, *p* = 0.001), STAT1 (by 0.86-fold, F_5,12_ = 103.2, *p* < 0.0001), and STAT3 (by 0.93-fold, F_5,12_ = 39.93, *p* < 0.0001) compared to AG490, nifuroxazide, S3I-201, and STA-21. It was also confirmed in MDA-MB-468 cells (Fig. 3E and F).

In summary, chalcone-9 inhibited the tyrosine phosphorylation of JAK1, JAK2, STAT1, and STAT3 in the JAK-STAT signaling pathway. In addition, it proved to be the compound with the highest potency compared to other commonly used JAK and STAT3 inhibitors.

### Chalcone-9 binds to the kinase domains of JAK1 and JAK2

Based on the investigation of the protein expression level in Fig. 3, we examined whether the structure of chalcone-9 can stably interact with JAK1 and JAK2 to inhibit their activation using the in silico molecular docking analysis.

**Fig. 4:**
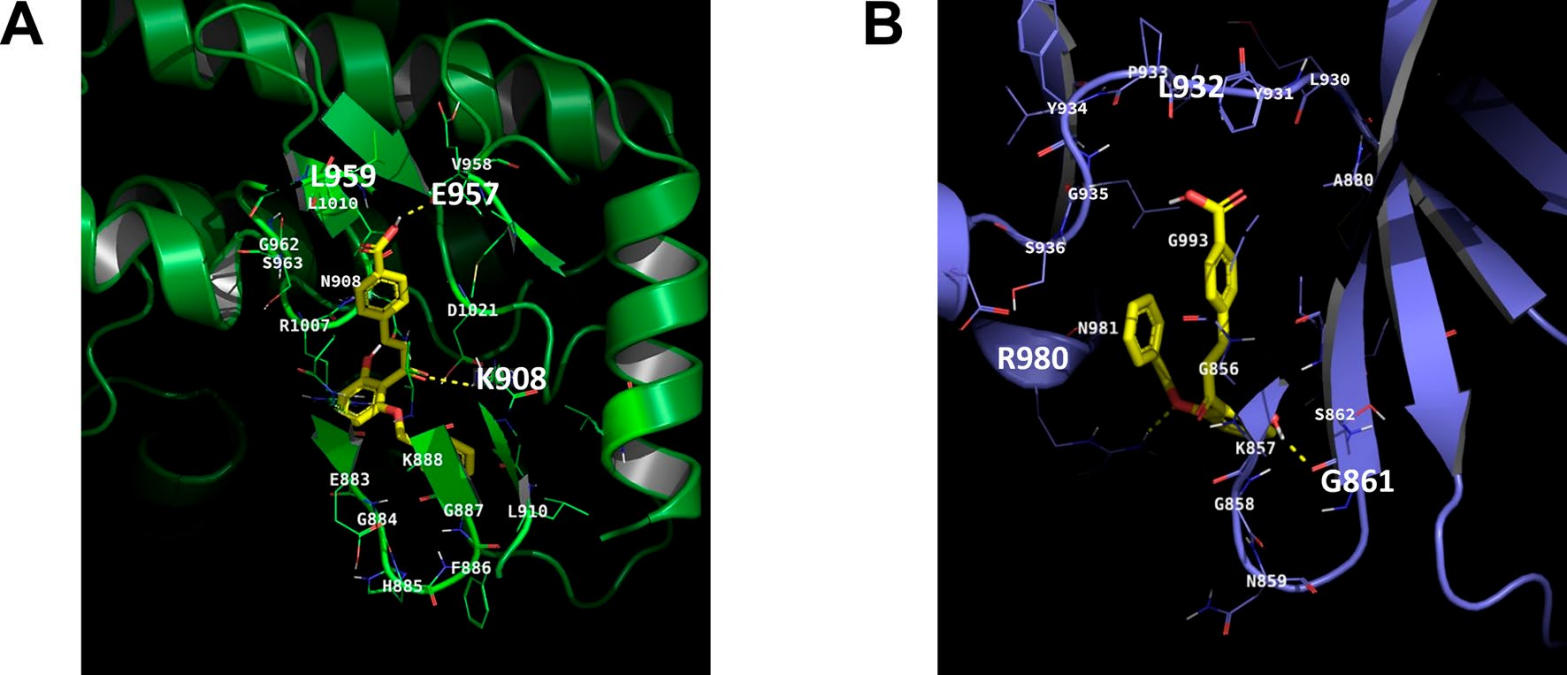
Chalcone-9 binds to the kinase domains of JAK1 and JAK2. Docking results from autodock-VINA are shown for chalcone-9 co-crystallized with (**A**) JAK1 (green), and (**B**) JAK2 (blue) protein. The yellow compound represents the structure of chalcone-9, and the dashed lines indicate hydrogen bonds. JAK; janus kinase

First, it is revealed that the benzoic acid group of chalcone-9 is oriented toward the kinase hinge region of JAK1, forming a hydrogen bond with Glu 957 (Fig. 4A). This group is positioned close to the backbone atoms of Leu 959, suggesting the potential for additional hydrogen bond formation. The carbonyl group of chalcone-9 forms a hydrogen bond with Lys 908, which is the same type of interaction that the phosphate group of ADP forms with Lys 908 in JAK1 [24]. Furthermore, the Gly residues in the β-sheet of JAK1 create a space for the benzene group of chalcone-9, enabling chalcone-9 to maintain a stable conformation with JAK1. Based on this docking analysis result, it can be inferred that chalcone-9 interacts stably with the kinase domain of JAK1.

Similarly, the benzoic acid group of chalcone-9 is also directed toward the kinase hinge region of JAK2 (Fig. 4B). Although no direct hydrogen bond formation is observed, the benzoic acid group is located close to the backbone atoms of Leu 932, suggesting the possibility of additional hydrogen bonding. The oxygen atoms on both sides of the benzene ring in the chalcone-9 act as hydrogen bond donors and acceptors, forming hydrogen bonds with Gly 861 in the β-sheet region of JAK2 and Arg 980 in the solvent-accessible area. This interaction is similar to that observed with fedratinib, another JAK2 inhibitor [25]. Due to these interactions with JAK2, the benzene ring of chalcone-9 is positioned in the opposite direction compared to its interaction with JAK1. In this orientation, the benzoic acid group and benzene ring of chalcone-9 are positioned close, contributing to stabilizing its internal conformation through hydrophobic interactions within JAK2. The docking scores of chalcone-9 with JAK1 and JAK2 are −9.21 kcal/mol and −8.02 kcal/mol, respectively. These values indicate that chalcone-9 can effectively dock with both JAK1 and JAK2. These overall molecular docking results indicate that chalcone-9 stably binds to both JAK1 and JAK2.

### Chalcone-9 inhibits the mRNA expression of STAT1 and STAT3 target genes, suppressing tumor migration and cell cycle progression

**Fig. 5:**
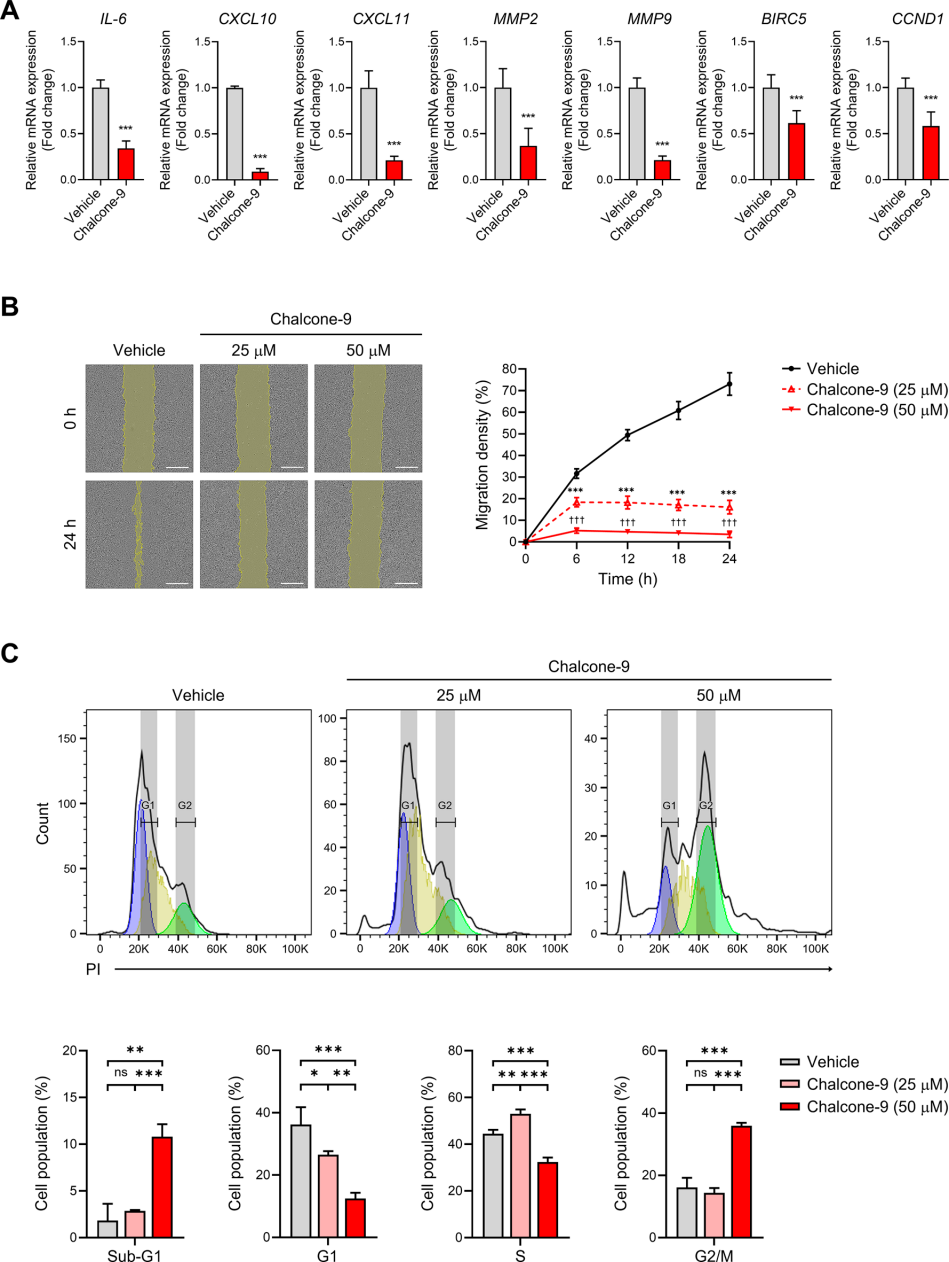
Chalcone-9 inhibits the expression of STAT1 and STAT3 target genes, suppressing tumor cell migration and induction of G2/M cell cycle arrest. **A** qRT-PCR analysis of IL-6, CXCL10, CXCL11, MMP2, MMP9, BIRC5, and CCND1 mRNA expression in vehicle or chalcone-9 (50 μM, 24 h)-treated MDA-MB-231 cells. Data were shown as mean ± SD (*n* = 9). Statistical significance is indicated as *p < 0.05, **p < 0.01, ***p < 0.001; compared with the vehicle-treated group. All statistical analyses were performed using unpaired Student’s *t*-test. **B** cell images of MDA-MB-231 cells treated with vehicle and chalcone-9 for 24 h in wound healing assay with a magnification of 40X (left). Scale bar, 500 μm. Quantification of migration density after treatment with chalcone-9, as shown in left cell images (right). The wound area was analyzed using ImageJ software. Data were shown as mean ± SD (*n* = 8). Statistical significance is indicated as *p < 0.05, **p < 0.01, ***p < 0.001; chalcone-9 (25 μM) vs vehicle-treated group, †*p* < 0.05, ††*p* < 0.01, †††*p* < 0.001; chalcone-9 (50 μM) vs vehicle-treated group at each time point. All statistical analyses were performed using one-way ANOVA followed by Dunnett’s post hoc test. **C** flow cytometry analysis with PI staining showed the percentage of each cell cycle phase in MDA-MB-231 cells treated with vehicle and chalcone-9 for 24 h (upper). The percentages of MDA-MB-231 cells in sub-G1, G1, S, and G2/M phases are shown (bottom). Data were shown as mean ± SD (*n* = 3). Statistical significance is indicated as *p < 0.05, **p < 0.01, ***p < 0.001, ns, not significant; compared with the vehicle-treated group. All statistical analyses were performed using one-way ANOVA followed by Tukey’s post hoc test. BIRC5; baculoviral IAP repeat containing 5, CCND1; cyclin D1, CXCL10; C-X-C motif chemokine ligand 10, CXCL11; C-X-C motif chemokine ligand 11, IL-6; interleukin-6, JAK; janus kinase, MMP2; matrix metallopeptidase 2, MMP9; matrix metallopeptidase 9, SD; standard deviation

Considering the activated STAT1 and STAT3 function as transcription factors [8, 26, 27], the inhibitory effect of chalcone-9 on STAT1 and STAT3 activation is expected to affect the transcription of their target genes ultimately. Therefore, we harvested cells after chalcone-9 treatment and verified the mRNA expression levels of STAT1 and STAT3 target genes through qRT-PCR. The mRNA expression levels of IL-6 (*t*_16_ = 17.23, *p* < 0.0001), CXCL10 (*t*_16_ = 73.6, *p* < 0.0001), and CXCL11 (*t*_16_ = 14.44, *p* < 0.0001) (which promote tumor-associated inflammation and immune escape in cancer), MMP2 (*t*_16_ = 6.774, *p* < 0.0001) and MMP9 (*t*_16_ = 20.86, *p* < 0.0001) (which are involved in tumor cell migration and invasion), as well as BIRC5 (*t*_16_ = 5.977, *p* < 0.0001) and CCND1 (*t*_16_ = 6.824, *p* < 0.0001) (which contribute to tumor cell proliferation), were significantly reduced following chalcone-9 treatment (Fig. 5A).

This suppression of STAT1- and STAT3-target genes suggests that chalcone-9 may disrupt tumor cell functions related to those genes [8, 10, 27]. Accordingly, we explored whether chalcone-9 led to the impairment of tumor cell function. First, it was observed through wound healing assay that chalcone-9 significantly reduced tumor cell migration. After scratching and incubation for 24 h (F_2,21_  = 838.0), comparison with the vehicle group showed that chalcone-9 at 25 μM reduced migration density by over 50% (*p* < 0.0001), and chalcone-9 at 50 μM further reduced it by approximately 70% (*p* < 0.0001) (Fig. 5B). Together, cell cycle analysis was performed through PI staining and flow cytometry. The G2/M phase cell population in the vehicle-treated group was 16.1% ± 3.1%, which significantly increased to 35.9% ± 0.9% when treated with 50 μM of chalcone-9 (F_2,6_  = 100.1, *p* < 0.0001) (Fig. 5C). This indicates that chalcone-9 triggered cell cycle arrest at the G2/M phase.

These results show that the inhibitory effect of chalcone-9 on the JAK-STAT signaling pathway suppresses the mRNA expression of STAT1 and STAT3 target genes, which play an important role in tumor cell function. This ultimately reduces tumor cell migration and induces cell cycle arrest.

### Chalcone-9 induces apoptosis of breast cancer cells

**Fig. 6:**
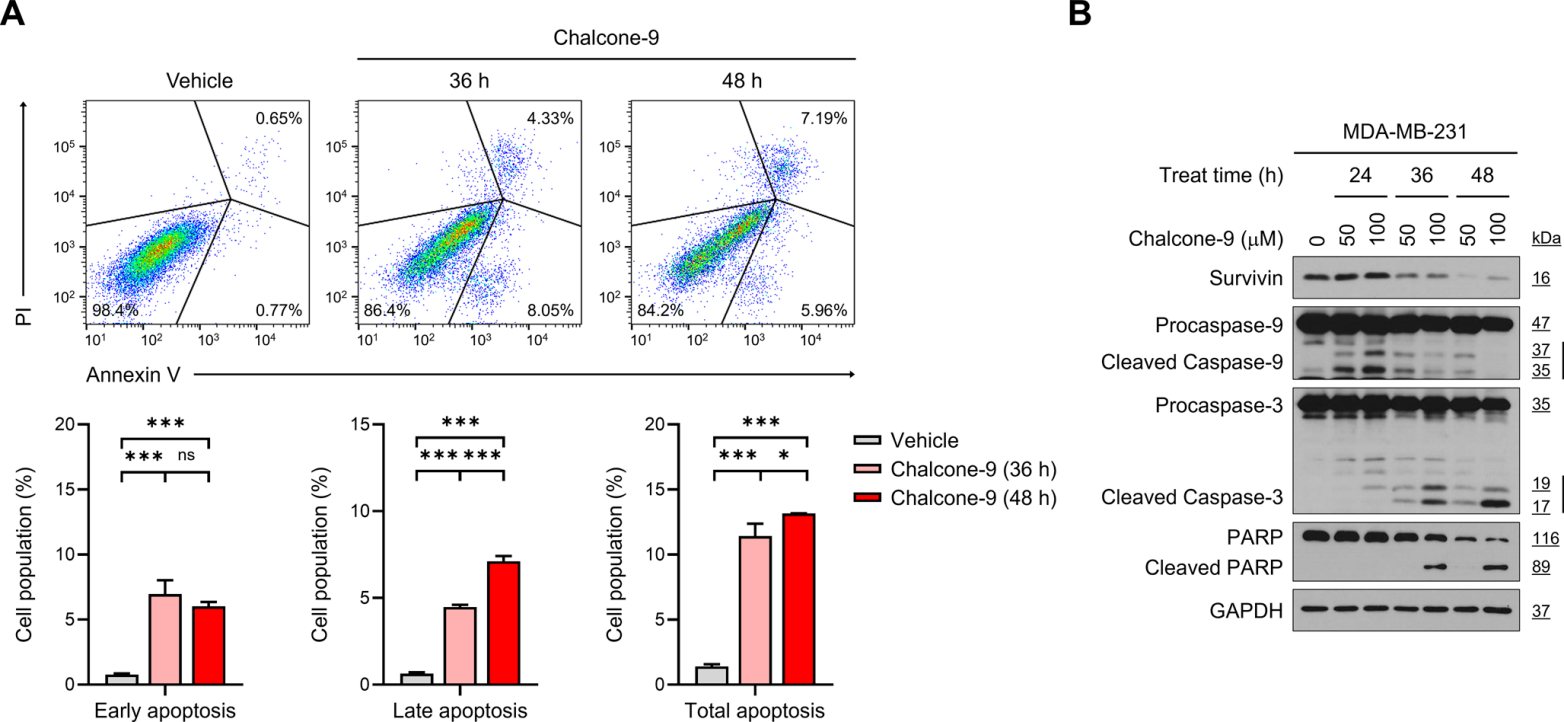
Chalcone-9 induces apoptosis of breast cancer cells. **A** flow cytometry analysis with Annexin V-PI staining was performed to evaluate the population of apoptotic cells in MDA-MB-231 cells treated with vehicle and 50 μM chalcone-9 for 36 h and 48 h. Flow cytometry quadrant histogram showed the changes in the percentage rate of early apoptosis (PI−/AnnexinV+), late apoptosis (PI+/AnnexinV+), and live cells (PI−/AnnexinV−). The percentage of apoptotic cells in MDA-MB-231 cells is shown (bottom). Data were shown as mean ± SD (*n* = 3). Statistical significance is indicated as *p < 0.05, **p < 0.01, ***p < 0.001, ns, not significant; compared with the vehicle-treated group. All statistical analyses were performed using one-way ANOVA followed by Tukey’s post hoc test. **B** Western blot analysis for survivin, caspase-9, caspase-3, PARP, and GAPDH in MDA-MB-231 cells treated with chalcone-9. GAPDH; glyceraldehyde-3-phosphate dehydrogenase, PARP; poly(ADP-ribose) polymerase, SD; standard deviation

As shown in Fig. 5C, chalcone-9-induced cell cycle arrest is expected to result in apoptosis of breast cancer cells. Therefore, we assessed the population of apoptotic cells following chalcone-9 treatment through FITC Annexin-V/PI staining and flow cytometry in MDA-MB-231 cells. After 36 h of treatment with 50 μM chalcone-9, the population of total apoptotic cells significantly increased compared to the control (1.4% ± 0.2% to 11.4% ± 0.95%, *p* < 0.0001), with a higher proportion of early apoptotic cells. After 48 h of chalcone-9 treatment, the population of total apoptotic cells further increased compared to the 36-h treatment (11.4% ± 0.95% to 13.2% ± 0.02%, *p* < 0.0001), with a more significant proportion of late apoptotic cells (The comparisons of total apoptosis; F_2,6_  = 389.3) (Fig. 6A). In addition, we examined the expression levels of intracellular proteins involved in cell survival or apoptosis. The protein level of survivin, an inhibitor of the apoptosis protein family, was reduced by chalcone-9 after treatment for 36 h and 48 h. As apoptosis was initiated with chalcone-9 treatment, caspase-9 and caspase-3 were sequentially activated. Specifically, PARP was cleaved after treatment with 100 μM of chalcone-9 for 36 and 48 h (Fig. 6B). In conclusion, these results suggest that chalcone-9 serves as an anti-cancer drug capable of triggering apoptotic cell death.

## Discussion

In this study, we identified the potential of chalcone-9 as a novel JAK-STAT pathway inhibitor and anti-cancer drug for aggressive TNBC cells. We discovered that chalcone-9 showed the most significant inhibitory effect on the JAK-STAT pathway and cytotoxicity against various human breast cancer cell lines. Notably, MDA-MB-231 and MDA-MB-468 cells, representative of TNBC subtypes, exhibited greater sensitivity to chalcone-9 compared to luminal breast cancer subtypes, such as MCF7 and T47D. Also, we concisely examined the impact of chalcone-9 on the JAK-STAT signaling pathway in MDA-MB-231 and MDA-MB-468 cells. We observed a significant reduction in the phosphorylation of JAK1, JAK2, STAT1, and STAT3 in a dose-dependent manner. Time-course experiments exposed persistent inhibitory effects, with 25 μM chalcone-9 demonstrating optimal efficacy while maintaining cell viability. Chalcone-9 surpassed common JAK and STAT inhibitors in interfering with the activation of JAK1, JAK2, STAT1, and STAT3 in MDA-MB-231 and MDA-MB-468 cells, emphasizing its potential as a highly effective inhibitor of the JAK-STAT signaling pathway. Based on these results, it is inferred that chalcone-9 acts as a potent inhibitor primarily targeting JAK1 and JAK2, the upstream regulators of STAT1 and STAT3 within the JAK-STAT signaling pathway.

Because TNBC with poor prognosis lacks the expression of ER, PR, and HER2, conventional targeted therapies, including trastuzumab, pertuzumab, and lapatinib, are ineffective against TNBC. Furthermore, tamoxifen treatment is limited by the ability of cancer cells to acquire resistance. Therefore, research into the discovery of new treatments and mechanisms for breast cancer is still necessary. Our previous research confirmed the molecular mechanism driving tamoxifen-resistant breast cancer cells, revealing the crucial role of STAT3 signaling in tamoxifen resistance [28,29]. Considering these findings, the inhibitory effect of chalcone-9 on the JAK-STAT pathway, including STAT3, implies that chalcone-9 is a very prospective breast cancer treatment.

We treated breast cancer cell lines with the well-known tamoxifen, which has already been FDA-approved for breast cancer treatment, and AG490, which is used as a JAK2 selective inhibitor, to compare the cytotoxicity of chalcone-9. We found that while chalcone-9 exhibits weaker cytotoxicity compared to tamoxifen, it demonstrates selective cytotoxic effects toward TNBC cells, which is a key advantage over non-specific cytotoxic agents. Unlike tamoxifen that indiscriminately target both cancerous and normal cells, chalcone-9‘s preferential activity against aggressive TNBC cells suggests its potential as a targeted therapeutic candidate. In the case of AG490, it showed much weaker cytotoxicity compared to chalcone-9 and tamoxifen.

Since JAK kinases are upstream kinases that activate STATs as ultimate transcription factors, it is expected that chalcone-9 inhibits JAK1 and JAK2, thereby suppressing STAT1 and STAT3. It was reported that licochalcone B, a chalcone derivative obtained from the *Glycyrrhiza inflata* plant, was predicted to interact with the ATP-binding pocket of JAK2 by computational docking modeling [30]. Therefore, we conducted an in silico molecular docking analysis to gain an understanding of the interaction between chalcone-9 and JAK1/JAK2 proteins. Chalcone-9 forms hydrogen bonds with both JAK1 and JAK2, and its conformation is stably docked in distinct orientations within each protein, suggesting its potential activity. To further strengthen the evidence, it is necessary to investigate the binding of chalcone-9 to JAK1 and JAK2 not only through virtual interaction analysis based on the chalcone-9 structure but also through an in vitro binding assay [31]. However, licochalcone B reduced the phosphorylation of STAT3 at both Tyr705 and Ser727. The relationship between Tyr705 phosphorylation and Ser727 phosphorylation of STAT3 remains unclear, with several reports indicating that different upstream kinases are involved in each phosphorylation event [32]. Furthermore, it is known that they mediate distinct downstream cellular functions [33, 34]. We believe that chalcone-9s ability to specifically decrease Tyr705 phosphorylation of STAT3 is advantageous in preventing side effects.

We explored the downstream effects of chalcone-9 on the reduced expression of STAT1 and STAT3 target genes, such as IL-6, CXCL10, CXCL11, MMP2, MMP9, BIRC5, and CCND1. Chalcone-9 hindered the intrinsic functions of tumor cells, demonstrating a substantial decrease in tumor cell migration and induction of cell cycle arrest at the G2/M phase. The increase in apoptotic cell populations, reduction in survivin protein levels, and successive activation of caspase-9 and caspase-3, coupled with PARP cleavage, provided evidence of chalcone-9-induced apoptosis. Because of the high cytotoxic effect of chalcone-9, there are concerns that it may have adverse effects on normal tissue when applied clinically. Alternatively, there may be concerns that it exhibits weaker anti-cancer activity compared to previously reported chalcone derivatives. Recent studies have explored the anti-cancer effects of chalcone derivatives, including TNBC, with some compounds showing activity at lower concentrations than chalcone-9 but also exhibiting non-specific cytotoxicity, which can lead to severe side effects [19]. However, chalcone-9 demonstrates selective anti-cancer activity against TNBC, setting it apart from other derivatives. Although it has been reported that chalcone derivatives exhibit stronger cell growth inhibition in TNBC compared to non-TNBC [35], we deem that their study focused only on the cell death mechanisms induced by these compounds while insufficiently exploring their direct targets or effects on intracellular signaling mechanisms. In our study, we present the JAK-STAT pathway inhibition effects of chalcone-9, along with its interaction with JAK1 and JAK2. Going et al. (2018) conducted a compelling proteomic analysis of a chalcone derivative that exhibited higher cytotoxicity in TNBC cells than in normal cells [36]. Their proteomics-based screening revealed an increase not only in cell death-related proteins but also in proteins associated with the major histocompatibility complex class I (MHC-I) pathway. This suggests that these chemicals may exert enhanced efficacy against TNBC cells through immune stimulation, making their study particularly intriguing. We believe that incorporating similar approaches into our future research could yield valuable insights.

Specific verification is also needed in our further study. The advantage of chalcone-9 lies in the convenience of modifying the chalcone derivative [18]. We have previously identified anti-melanogenic activity in one of our synthesized chalcone derivatives and successfully optimized its structure to enhance efficacy [22]. Building on this experience, we aim to further improve the anti-cancer activity of chalcone-9 while maintaining its selective potency against TNBC. In further studies, the well-established anti-cancer efficacy of chalcone-9 needs to be validated through in vivo animal experiments [19].

Recent clinical trials have explored the efficacy of JAK inhibitors in treating triple-negative breast cancer (TNBC). A notable phase II study investigated ruxolitinib, a selective JAK1/2 inhibitor, in patients with metastatic TNBC [37]. Despite the drug’s ability to reduce levels of phosphorylated STAT3, none of the 21 participants exhibited a clinical response to the therapy. These findings indicate that direct JAK inhibition with agents like ruxolitinib may not yield significant clinical benefits in TNBC. This suggests that JAK2 inhibitors may not be sufficiently effective as monotherapy for TNBC. Therefore, further research is needed on combination therapies involving JAK2 inhibitors and other anti-cancer agents for TNBC treatment.

Recently, a study reported that JAK2 signaling is involved in paclitaxel resistance in TNBC [38]. In this study, treatment with a JAK1/2 inhibitor restored drug sensitivity in certain patient-derived xenograft (PDX) models exhibiting paclitaxel resistance. These findings indicate that JAK inhibitors hold great potential for various applications in TNBC treatment.

Moreover, clinical trials involving chalcone derivatives have been limited. This is because these compounds are primarily studied at the preclinical stage [39]. Therefore, further research and clinical trials are necessary to evaluate the clinical applicability of chalcone derivatives.

In conclusion, we demonstrated that chalcone-9 inhibits the JAK-STAT signaling pathway in breast cancer cells, leading to the downregulation of target gene mRNA expression, suppression of tumor cell functions, and ultimately, induction of apoptosis. These findings highlight the potential of chalcone-9 as a promising anti-cancer agent.

## Electronic supplementary material

Below is the link to the electronic supplementary material.

Supplementary Material 1

## Data Availability

No datasets were generated or analysed during the current study.

## Abbreviations

ANOVA: Analysis of Variance
ATCC: American Type Culture Collection
BIRC5: Baculoviral IAP Repeat Containing 5
CCK-8: Cell Counting Kit-8
CCND1: Cyclin D1
CXCL10: C-X-C motif chemokine ligand 10
CXCL11: C-X-C motif chemokine ligand 11
DMEM: Dulbecco’s Modified Essential Medium
DMSO: Dimethyl Sulfoxide
EDTA: Ethylenediaminetetraacetic Acid
ER: Estrogen Receptor
FBS: Fetal Bovine Serum
FDA: Food and Drug Administration
FITC: Fluorescein Isothiocyanate
GAPDH: Glyceraldehyde-3-Phosphate Dehydrogenase
HER2: Human Epidermal Receptor 2
IL-6: Interleukin-6
JAK: Janus Kinase
MMP2: Matrix Metallopeptidase 2
MMP9: Matrix Metallopeptidase 9
PARP: Poly(ADP-ribose) Polymerase
PBS: Phosphate-Buffered Saline
PDX: Patient-Derived Xenograft
PI: Propidium Iodide
PMSF: Phenylmethylsulfonyl Fluoride
PR: Progesterone Receptor
pS: Phosphoserine
pY: Phosphotyrosine
qRT-PCR: Quantitative Real-Time Polymerase Chain Reaction
SD: Standard Deviation
SDS: Sodium Dodecyl Sulfate
STAT: Signal Transducer and Activator of Transcription
TBS: Tris-Buffered Saline
TNBC: Triple-Negative Breast Cancer
TYK: Tyrosine Kinase
