# Supplementary material for: Chalcone-9: a novel inhibitor of the JAK-STAT pathway with potent anti-cancer effects in triple-negative breast cancer cells
Source: Pharmacol Rep. 2025 Apr 9;77(3):761–74. doi: 10.1007/s43440-025-00721-w (PMC12066378; doi:10.1007/s43440-025-00721-w)
Supplement: Supplementary file 1 — Supplementary Material 1 [file 43440_2025_721_MOESM3_ESM.pdf]

Supplementary Information

Chalcone-9: a novel inhibitor of the JAK-STAT pathway with potent anti-cancer effects in triple-negative breast cancer cells

Song-Hee Lee[1†], Haeri Lee[1,2], Yong-Jin Kwon[3], Seul-Ki Kim[1], Eun-Bi Seo[1,2], Jie Ohn Sohn[4], Byung-Hak Kim[5], Jung-Youl Park[6], and Sang-Kyu Ye[1,2,4,7,8*]

[1]Department of Biomedical Sciences and Pharmacology, Seoul National University College of Medicine, Seoul, 03080, Republic of Korea

[2]Ischemic/Hypoxic Disease Institute, Seoul National University College of Medicine, Seoul, 03080, Republic of Korea

[3]Department of Cosmetic Science, Kyungsung University, Busan, 48434, Republic of Korea

[4]Wide River Institute of Immunology, Seoul National University, Hongcheon, Gangwon-do 25159, Republic of Korea

[5]Medience Co. Ltd., Chuncheon, Gangwon-do 24232, Republic of Korea

[6]Glocal University Project Group, Andong National University, Andong, Gyeongsangbuk-do 36729, Republic of Korea

[7]Biomedical Science Project (BK21PLUS), Seoul National University College of Medicine, Seoul, 03080, Republic of Korea

[8]Neuro-Immune Information Storage Network Research Center, Seoul National University College of Medicine, Seoul, 03080, Republic of Korea

*Corresponding author: Sang-Kyu Ye, Department of Biomedical Sciences and Pharmacology, Seoul National University College of Medicine, 103 Daehak-ro, Jongno-gu, Seoul 03080, Republic of Korea

Tel: +82-2-740-8281; Fax: +82-2-745-7996; E-mail: sangkyu@snu.ac.kr (S.K. Ye)

† First author

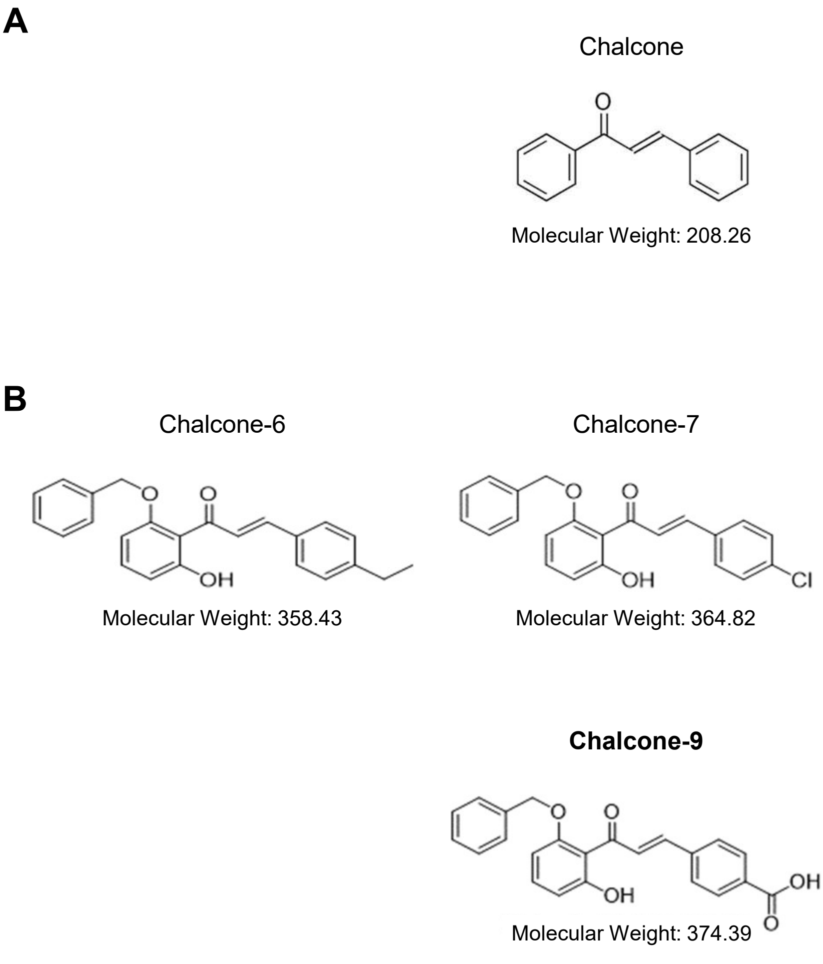

Fig. S1. Chemical structures of chalcone and its derivatives. (A) Structure of original chalcone (M.W. = 208.26). (B) Structures of chalcone-6 (M.W. = 358.43), chalcone-7 (M.W. = 364.82), chalcone-8 (M.W. = 360.40) and chalcone-9 (M.W. = 374.39) used in this study.

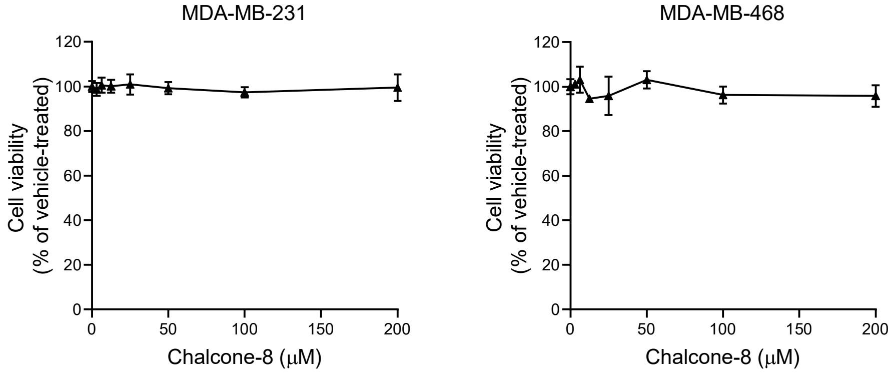

Fig. S2. Effects of chalcone-8 on cell viability in MDA-MB-231 and MDA-MB-468 cells. Cell viability was measured by CCK-8 assay in MDA-MB-231 and MDA-MB-468 cells. Cell viability is represented as % control compared with the vehicle-treated group. Data were shown as mean ± SD (n = 3).

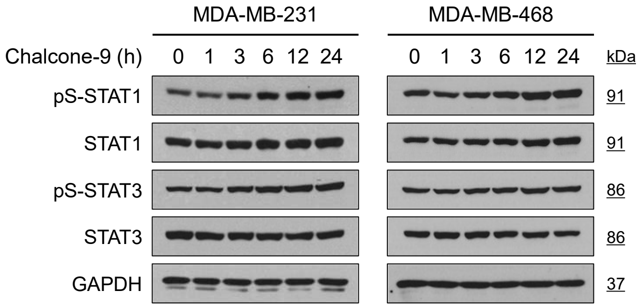

Fig. S3. Effects of chalcone-9 on serine phosphorylation of STAT1 and STAT3 in MDA-MB-231 and MDA-MB-468 cells. Western blot analysis of STAT1 and STAT3 serine phosphorylation following treatment with 25 μM chalcone-9 in a time-dependent manner.

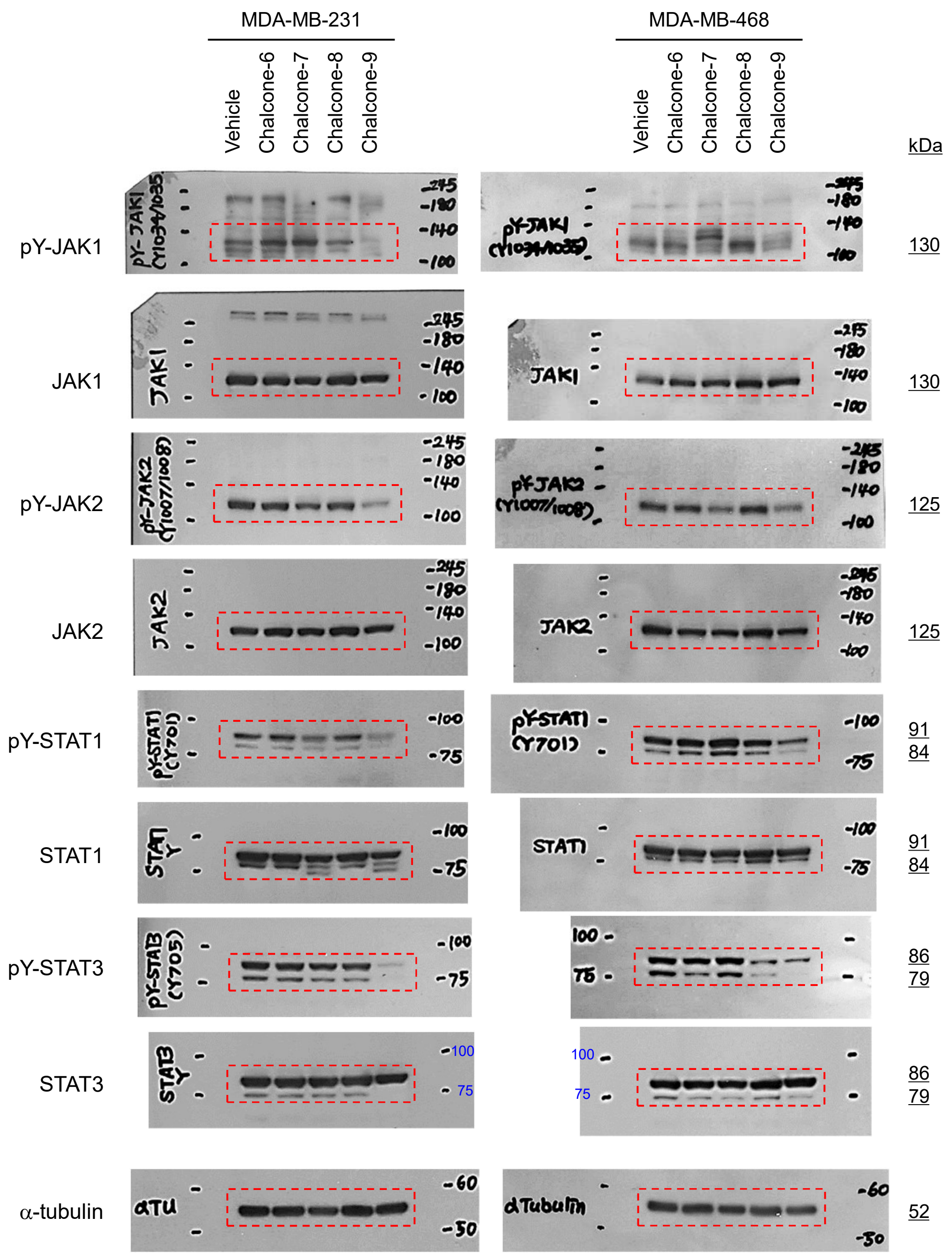

Fig. S4. Unprocessed western blot images corresponding to the representative immunoblots shown in Fig. 1A, depicting the labeling of pY-JAK1, JAK1, pY-JAK2, JAK2, pY-STAT1, STAT1, pY-STAT3, STAT3, and α-tubulin in vehicle or chalcone derivatives-treated MDA-MB-231 and MDA-MB-468 cells.

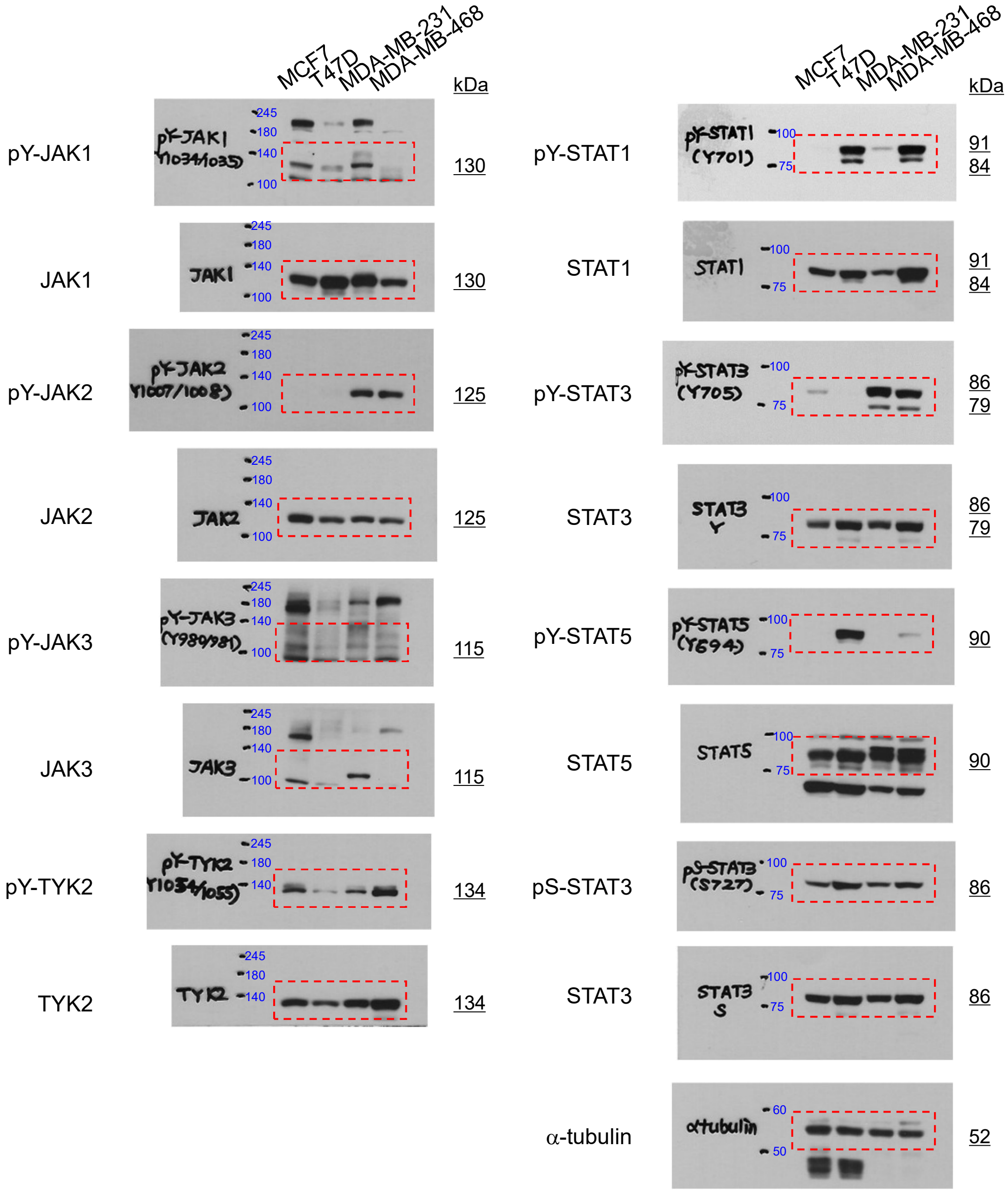

Fig. S5. Unprocessed western blot images corresponding to the representative immunoblots shown in Fig. 2B, depicting the labeling of pY-JAK1, JAK1, pY-JAK2, JAK2, pY-JAK3, JAK3, pY-TYK2, TYK2, pY-STAT1, STAT1, pY-STAT3, pS-STAT3, STAT3, pY-STAT5, STAT5, and α-tubulin in MCF7, T47D, MDA-MB-231, and MDA-MB-468 cells.

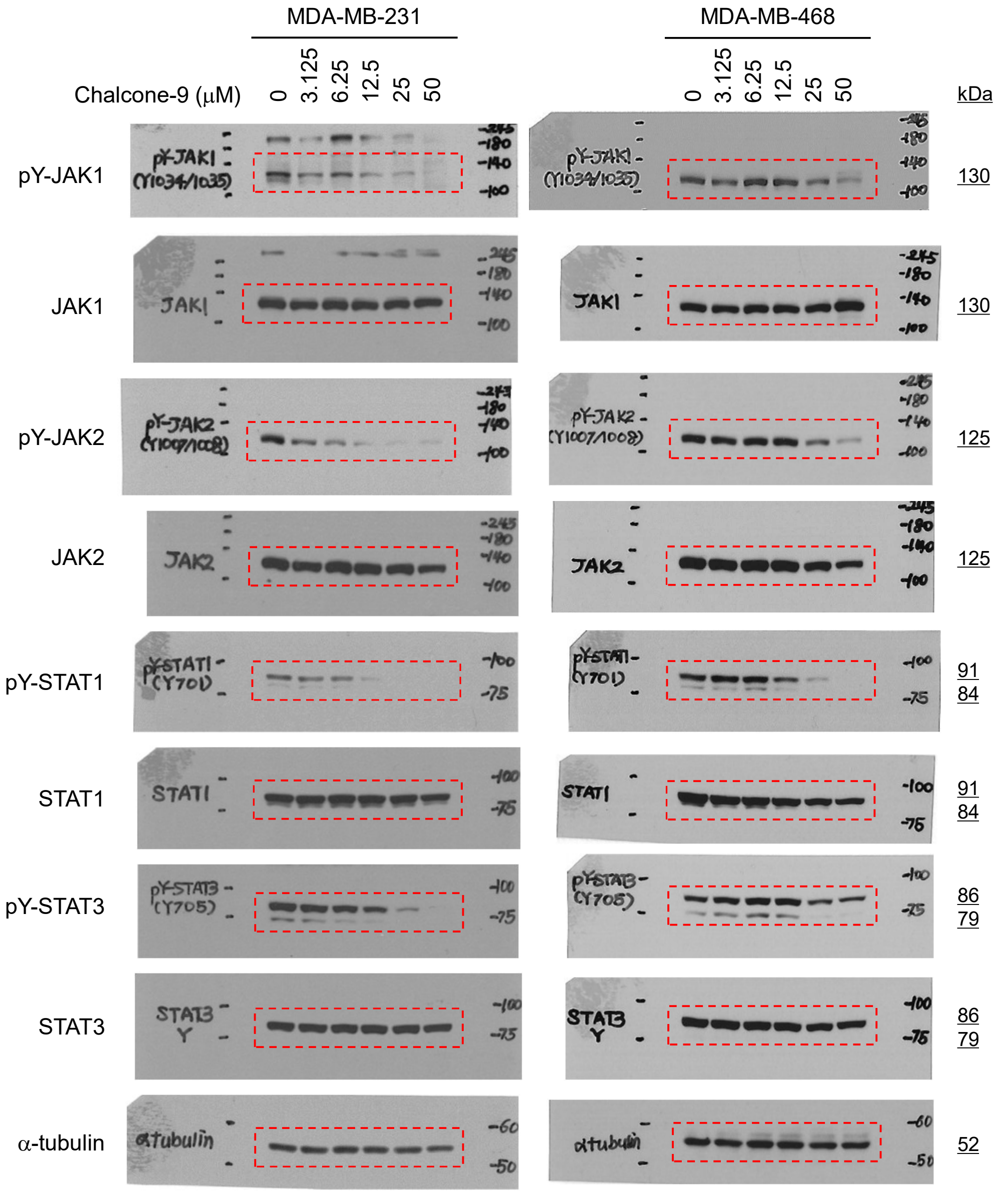

Fig. S6. Unprocessed western blot images corresponding to the representative immunoblots shown in Fig. 3A, depicting the labeling of pY-JAK1, JAK1, pY-JAK2, JAK2, pY-STAT1, STAT1, pY-STAT3, STAT3, and α-tubulin in MDA-MB-231 and MDA-MB-468 cells treated with chalcone-9 in a dose-dependent manner.

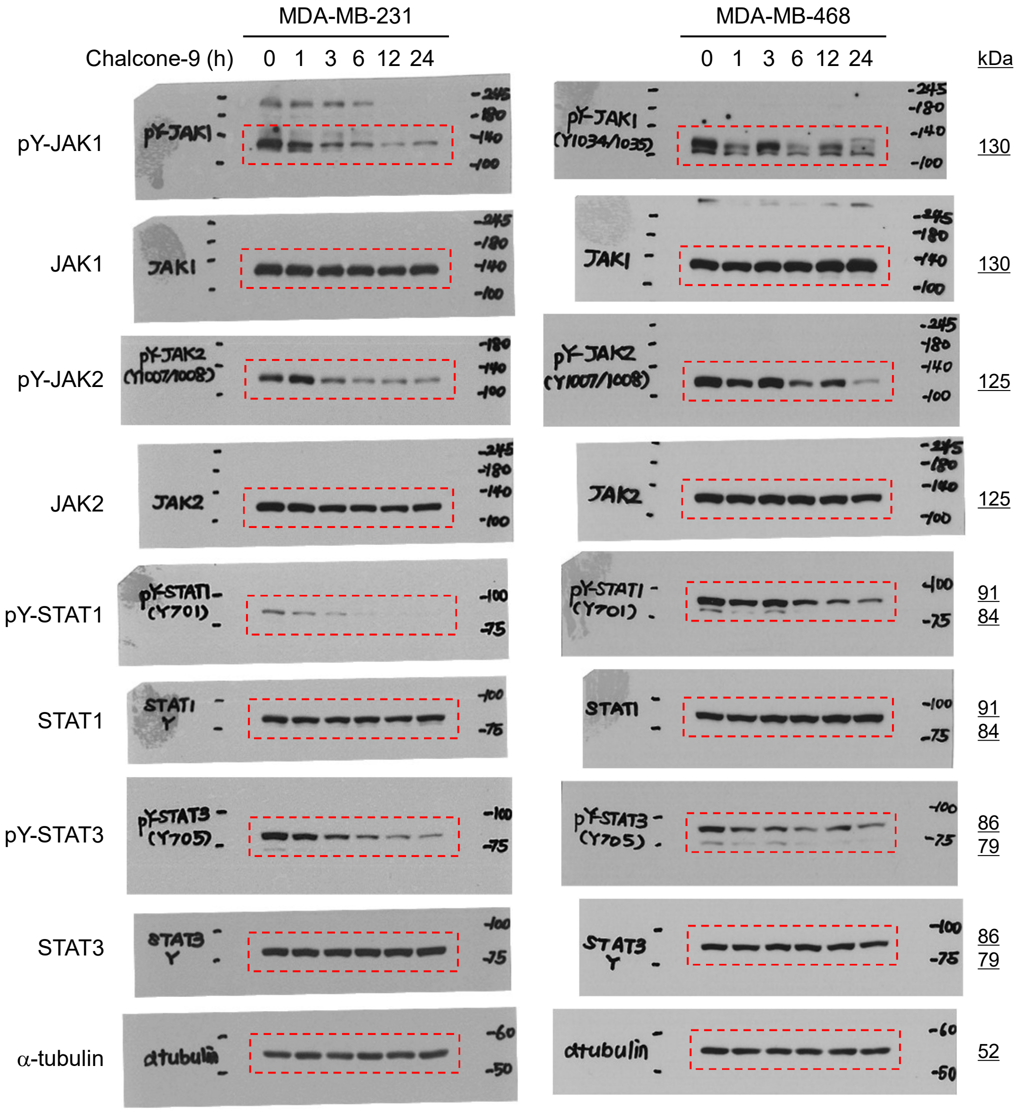

Fig. S7. Unprocessed western blot images corresponding to the representative immunoblots shown in Fig. 3C, depicting the labeling of pY-JAK1, JAK1, pY-JAK2, JAK2, pY-STAT1, STAT1, pY-STAT3, STAT3, and α-tubulin in MDA-MB-231 and MDA-MB-468 cells treated with chalcone-9 in a time-dependent manner.

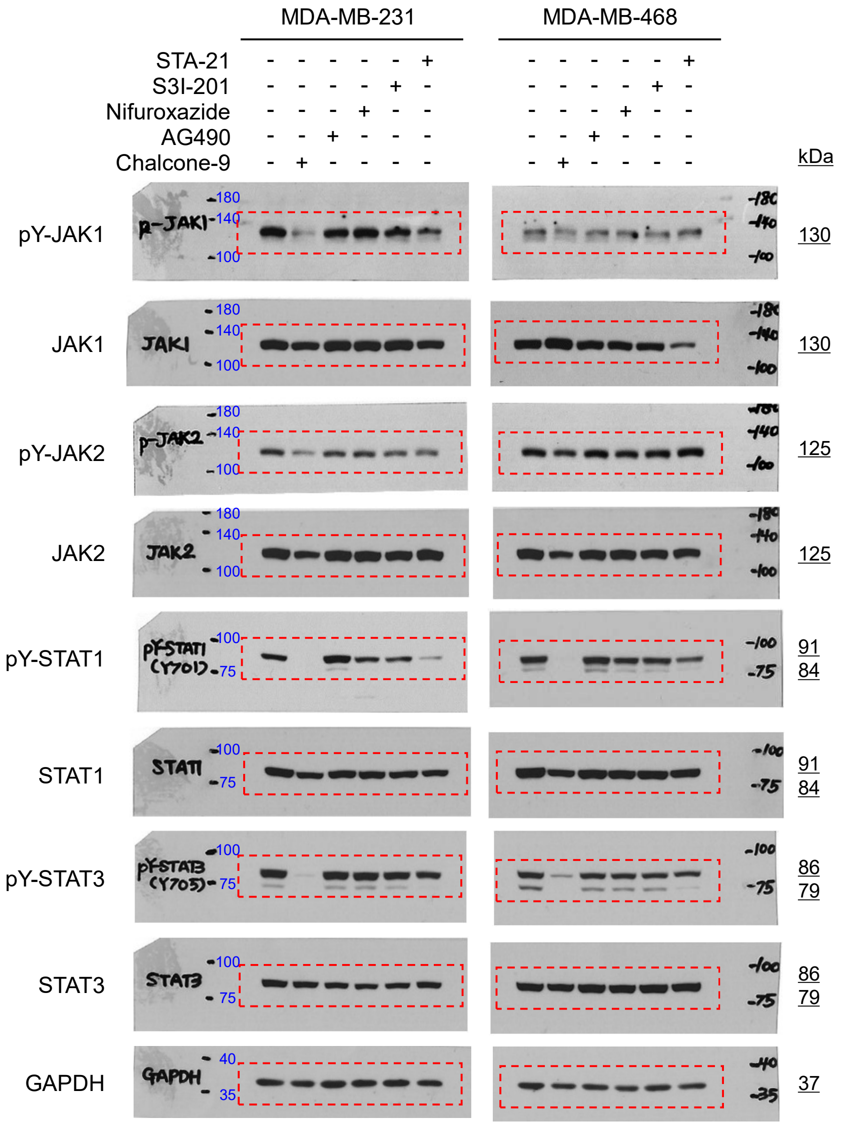

Fig. S8. Unprocessed western blot images corresponding to the representative immunoblots shown in Fig. 3E, depicting the labeling of pY-JAK1, JAK1, pY-JAK2, JAK2, pY-STAT1, STAT1, pY-STAT3, STAT3, and GAPDH in chalcone-9, AG490, nifuroxazide, S3I-201, or STA-21-treated MDA-MB-231 and MDA-MB-468 cells.

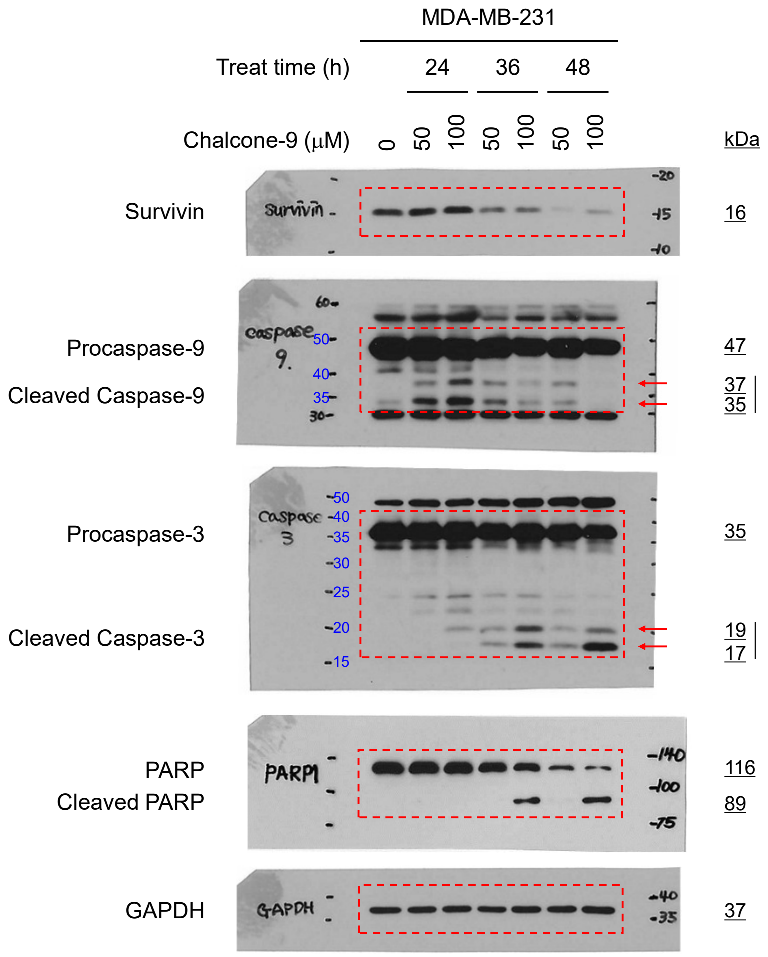

Fig. S9. Unprocessed western blot images corresponding to the representative immunoblots shown in Fig. 6B, depicting the labeling of survivin, caspase-3, caspase-9, PARP, and GAPDH in chalcone-9-treated MDA-MB-231 cells.

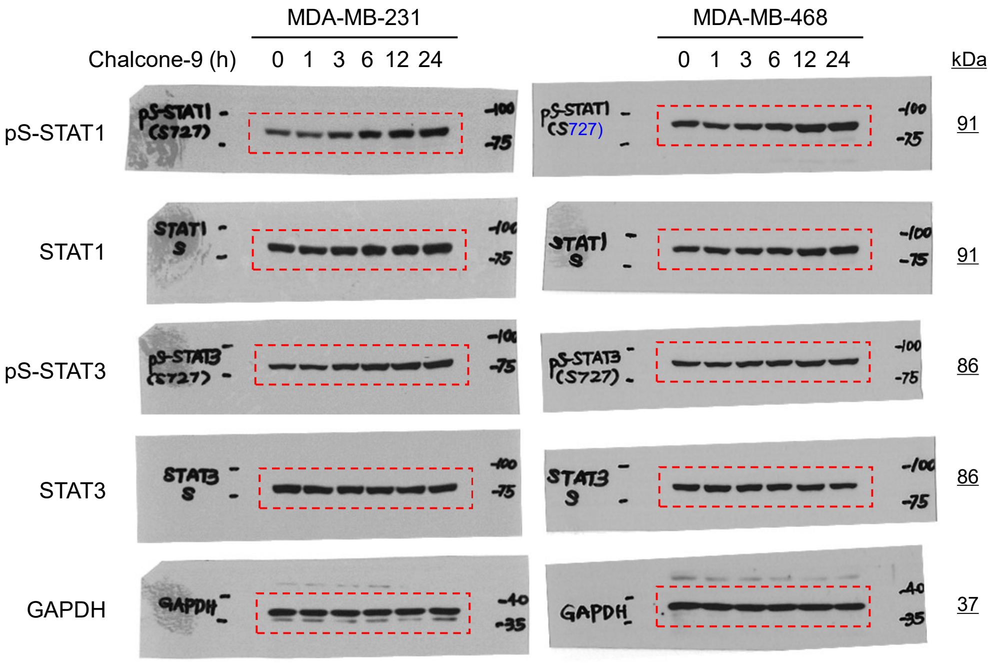

Fig. S10. Unprocessed western blot images from which representative immunoblots depicting the labeling of pS-STAT1, STAT1, pS-STAT3, STAT3, and GAPDH in MDA-MB-231 and MDA-MB-468 cells are shown in Figure S3.

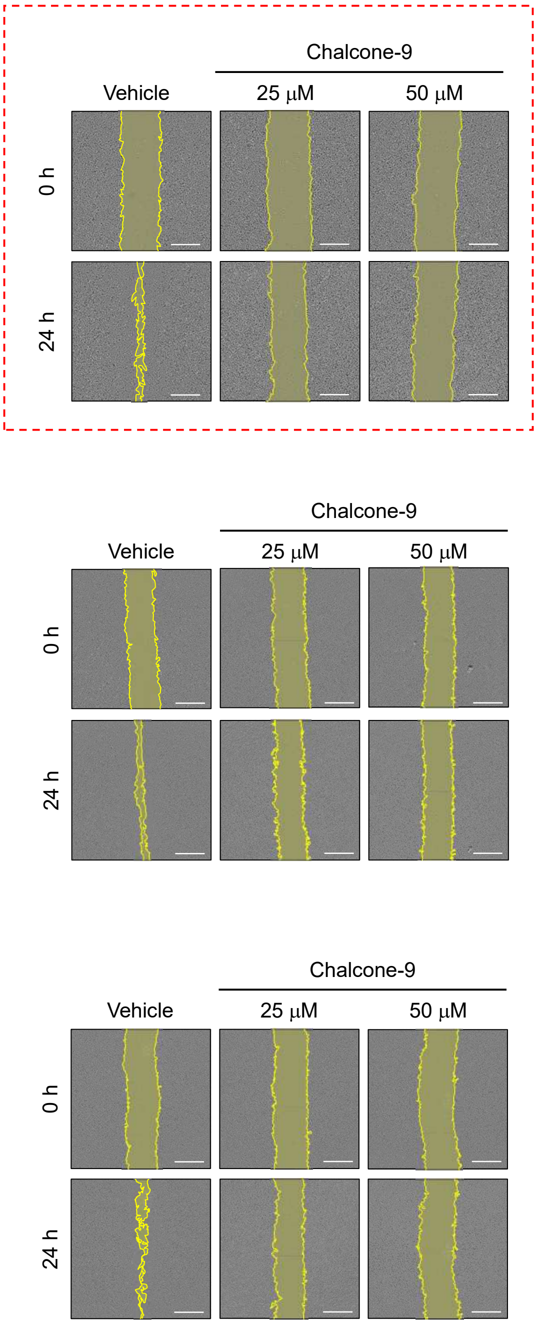

Fig. S11. Representative images of the wound healing assay shown in Fig. 5B (three images per experimental group). The representative images displayed in the main figure are indicated with a red box (top). Cell images of MDA-MB-231 cells treated with vehicle and chalcone-9 for 24 hours in wound healing assay with a magnification of 40X. Scale bar, 500 µm.
